# Cell type identification from single-cell transcriptomes in melanoma

**DOI:** 10.1186/s12920-021-01118-3

**Published:** 2021-11-17

**Authors:** Qiuyan Huo, Yu Yin, Fangfang Liu, Yuying Ma, Liming Wang, Guimin Qin

**Affiliations:** School of Computer Science and Technology, Xidian University, Xi’an, 710071 China

**Keywords:** Single-cell sequencing, Melanoma, Cell type, Cell marker, lncRNA

## Abstract

**Background:**

Single-cell sequencing approaches allow gene expression to be measured at the single-cell level, providing opportunities and challenges to study the aetiology of complex diseases, including cancer.

**Methods:**

Based on single-cell gene and lncRNA expression levels, we proposed a computational framework for cell type identification that fully considers cell dropout characteristics. First, we defined the dropout features of the cells and identified the dropout clusters. Second, we constructed a differential co-expression network and identified differential modules. Finally, we identified cell types based on the differential modules.

**Results:**

The method was applied to single-cell melanoma data, and eight cell types were identified. Enrichment analysis of the candidate cell marker genes for the two key cell types showed that both key cell types were closely related to the physiological activities of the major histocompatibility complex (MHC); one key cell type was associated with mitosis-related activities, and the other with pathways related to ten diseases.

**Conclusions:**

Through identification and analysis of key melanoma-related cell types, we explored the molecular mechanism of melanoma, providing insight into melanoma research. Moreover, the candidate cell markers for the two key cell types are potential therapeutic targets for melanoma.

## Background

Melanoma is a malignant tumor that develops from melanocytes and is considered a multifactorial disease caused by the interaction between genetic susceptibility factors and environmental exposure [1, 2]. Although the incidence of many cancers is declining, the incidence of melanoma is increasing [3, 4]. The prognosis of melanoma is proportionate to the depth of the tumor, which increases with time; thus, melanoma must be identified, detected, and treated in a timely manner [1]. Schomberg et al. [5] used RNA sequencing (RNA-seq) to profile luteolin-induced differentially expressed genes (DEGs) in 4 melanoma cell lines and found that luteolin-mediated growth inhibition may be mediated in melanoma cells through simultaneous action on multiple pathways. Mahata [6] proposed a clustering method to explore the subtypes of melanoma and breast cancer. Klinke et al. [7] developed an unsupervised feature extraction and selection strategy to capture functional plasticity separately tailored to breast cancer and melanoma.

The limitations of bulk RNA-seq data are that the molecular expression in a single cell is masked, and the cell heterogeneity in a sample is ignored. With the development of single-cell RNA sequencing (scRNA-seq) technology, scRNA-seq data analysis has been widely used in the study of different biological tissues, revealing the meanings of differential gene expression between cells [8–10] and researchers have begun to decipher the functional states of cancer cells at the single-cell level [11–14]. Various methods related to the life sciences have been applied in cancer research and have led to discoveries in cancer evolution, metastasis, treatment resistance and the tumor microenvironment [15, 16].

Compared with next generation RNA-seq data, there are more noise data and more dropouts in scRNA-seq data. There are several reasons for the dropout phenomenon [17]. Firstly, transcripts do not exist, so zero is an accurate representation of the state of a cell; secondly, the depth of sequencing is low, despite the existence of transcripts, it has not been reported. Thirdly, as part of library preparation, transcripts were not captured or failed to amplify. Some methods were proposed for imputing zeros. Lin et al. [18] introduced the Clustering through Imputation and Dimensionality Reduction(CIDR), which used a novel but very simple implicit imputation approach in a principled way in order to mitigate the impact of dropout values in scRNA-seq data. van Dijk et al. [19] developed the Markov affinity-based graph imputation of cells(MAGIC), to share information between similar cells through data diffusion to denoise the cell count matrix and fill in missing transcripts. Li et al. [20] introduced the scImpute to impute the dropout values in scRNA-seq data. Instead of eliminating the influence of dropout values to improve clustering accuracy, we attempted to amplify the influence of dropout values to explore the molecular mechanisms of melanoma.

In this study, we fully considered the characteristics of scRNA-seq data and identified a variety of cell types in melanoma cancer cells and a series of candidate cell markers for various cell types based on scRNA-seq gene and lncRNA expression data. Furthermore, by evaluation of each cell type, we identified and analyzed the key cell types associated with melanoma and then revealed the pathogenesis of melanoma, providing new insight into its diagnosis and prognosis.

## Methods

**Fig. 1:**
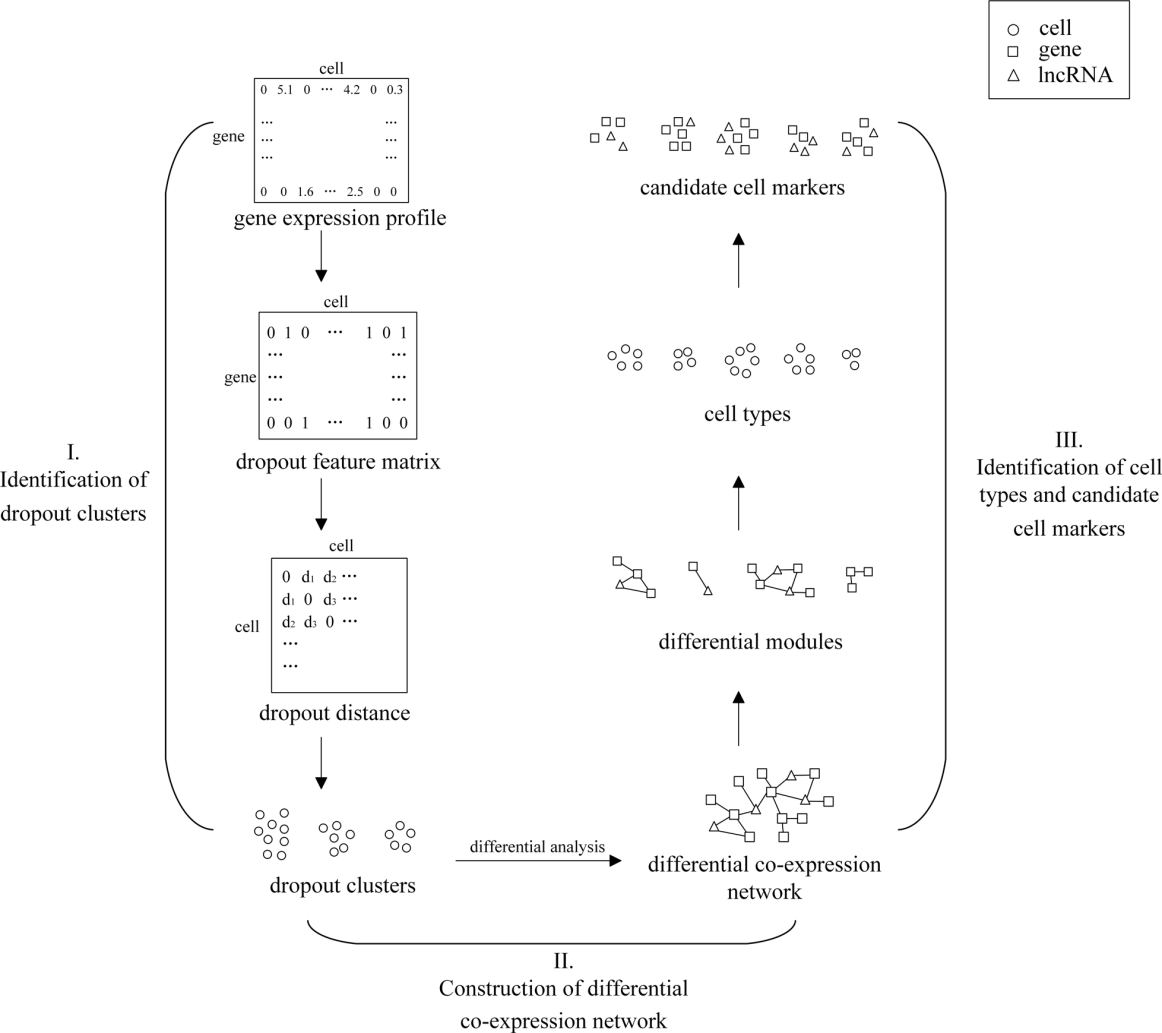
Schematic illustration of the scRNA-seq data-based cell type identification framework. (I) Identification of dropout clusters. First, the dropout information was extracted from the gene expression profile, and the dropout feature matrix was constructed. Then, the dropout distances between cells were calculated, and DBSCAN was performed to identify dropout clusters of cells. (II) Construction of the differential co-expression network. We performed differential analysis of the dropout rates and molecule expression levels and constructed a differential co-expression network based on the correlations between differential molecules. (III) Identification of cell types and candidate cell markers. We performed MCL on the differential co-expression network to identify differential modules. We then used these differential modules as new features to identify the cell types and further identified candidate cell markers for each cell type

In this paper, considering the characteristics of scRNA-seq data, we proposed a framework for cell type identification (Fig. 1). The framework consists of three parts: identification of dropout clusters, construction of the differential co-expression network, and identification of cell types and candidate cell markers.

### Molecular expression datasets

The expression profiles used in this experiment were extracted from the EXP0072 dataset in CancerSEA (http://biocc.hrbmu.edu.cn/CancerSEA/goDownload) [21], which was collated from the expression files from the GEO dataset GSE81383 [22]. scRNA-seq was applied to profile the transcriptomes of 307 single cells cultured from three biopsies of three different patients, who had BRAF/NRAS wild type, BRAF mutant/NRAS wild type and BRAF wild/NRAS mutant type metastatic melanoma. The expression profiles contain the expression values for 18,938 protein-coding genes and 15,626 lncRNAs.

### Known melanoma-related biomolecules

To analyze the correlation between each cell type pair, we collated known melanoma-related biomolecules from multiple public databases and published research results.

From the OMIM catalogue (https://omim.org/) [23], the COSMIC database (https://cancer.sanger.ac.uk/cosmic) [24] and a published study by Bailey et al. [25], we obtained 539, 63 and 24 known melanoma-related genes, respectively. A total of 580 known melanoma-related genes were obtained.

From the Lnc2Cancer 2.0 (http://www.bio-bigdata.net/lnc2cancer/) [26], LncRNADisease v2.0 (http://www.rnanut.net/lncrnadisease/) [27] and NONCODE v5.0 (http://www.noncode.org/) [28] data-bases, we obtained 24, 2,712 and 14 known melanoma-related lncRNAs, respectively. A total of 2,719 known melanoma-related lncRNAs were obtained.

In addition, we obtained a cell marker entity associated with melanoma from the CellMarker (http://biocc.hrbmu.edu.cn/CellMarker/) [29] database. This entry comprises data from cancer cells in peripheral blood and contains four cell marker genes, *MITF*, *MLANA*, *PMEL* and *TYR*. Schelker et al. [30] used scRNA-seq data to identify nine main cell types, and the four above mentioned genes (*MITF*, *MLANA*, *PMEL* and *TYR*) were used as cell markers for melanoma cell types.

### Data preprocessing

We used the EXP0072 dataset in CancerSEA [24], which contains the expression profiles of 18,938 protein-coding genes and 15,626 lncRNAs from 307 melanoma cancer cells.

First, we filtered the genes and lncRNAs with expression values of greater than 0 in fewer than 3 cells or average normalized expression values of less than 10^–5^, fitted the normal distribution to the genes (or lncRNAs) and cells, and removed cells in which significantly few genes were detected (FDR < 0.05). The resulting expression profile consisted of 307 cells, 15,488 genes and 8,524 lncRNAs.

Next, feature selection was performed for the genes and lncRNAs with non-log-transformed expression data using the M3Drop method in the M3Drop package [31].

### Feature selection

Cell clustering is dependent on the selection of genes, and traditional methods generally use the most variable genes as features. An important characteristic of scRNA-seq data is the high dropout rate, which usually accounts for more than half of the values in the expression matrix, and M3Drop [31] selects genes based on the dropout property.

M3Drop is based on the Michaelis–Menten equation, which is used to represent enzymatic reactions to fit the relationship between the average expression value and the dropout rate, as shown in (1):

1 $${P}_{dropout}=1-S/(K+S)$$

where *S* is the average expression level of the gene in all cells, *K* is the Michaelis constant, and *P*_*dropout*_ is the ratio of the dropout value of the gene to the expression of the gene in all cells, i.e., the dropout rate of the gene.

The parameter *K* in the Michaelis–Menten equation was used to calculate the specific *K*_*j*_ for gene j, and the global KM of all genes was fitted by the z-test. Then, the significant genes were selected as the result of feature selection by multiple testing corrections. The equation for calculating *K*_*j*_ is shown as (2):

2 $${K}_{j}=({P}_{j}*{S}_{j})/(1-{P}_{j} )$$

where *P*_*j*_, *S*_*j*_ and *K*_*j*_ are the corresponding *P*_*dropout*_, *S* and *K* values for gene j in (1).

Identification of dropout clusters.

To fully explore the dropout information in the single-cell expression data, we fuzzed specific gene expression values and highlighted dropout values. According to whether the gene expression in the cell was a dropout value, we binarized the gene expression data. In detail, all expression values greater than 0 in the gene expression profile were recorded as 1; otherwise, as 0. The matrix generated by binarizing the gene expression values was called the dropout feature matrix.

Next, we defined the dropout distance between cells based on the dropout features. There are a large number of zeros in scRNA-seq data, so we applied Manhattan distance to measure the distance between cells to avoid bias. Firstly, we calculated the Manhattan distance between each cell pair and then used the z-score to normalize the Manhattan distance to obtain the dropout distance between the cells.

Then, based on the dropout distance between the cells, we clustered cells with density-based spatial application of applications with noise (DBSCAN) [32], which can identify clusters with various shapes and sizes and effectively identify noise in cells. Before clustering, we visualized the data distribution and found it was based on the density distribution, and then compared to some other clustering algorithms(for example, SC3 and pcaReduce), DBSCAN is sensitive to noisy data while SC3 and pcaReduce often mistake noise for true structure, which means DBSCAN is more appropriate for our dataset[17]. We then defined dropout clusters as cell clusters obtained by cluster analysis based on the dropout distance.

Two hyperparameters should be determined in DBSCAN, one is the field radius *eps* that defines the field range, and the other is the minimum field point *MinPts* required for the sample to be defined as the core point. The parameter selection method is as follows:

Step 1 initialize *MinPts* as M_i_ and calculate the M_i_ distance range R for all samples. Given R and step size, say 0.001, calculate *eps* and the number of clusters k, and retain the *eps* that maximizes the silhouette coefficient [33] and set it as e_i_.

Step 2 assign e_i_ to *eps* and calculated the maximized the silhouette coefficient[33] of *MinPts*, marked as M_i_.

Step 3 update M_i_ and repeat Step 1 and Step 2. The parameters that maximized the silhouette coefficient[33] are set to be the input of DBSCAN.

### Construction of the differential co-expression network

To analyze the differences among dropout clusters, for each dropout cluster, we divided all the cells into two groups—cells belonging to the cluster and the remaining cells—and performed differential analysis of genes and lncRNAs from two aspects: the dropout rate and molecule expression value.

Differential dropout analysis. We calculated the dropout rate for all gene/lncRNA expression values for each cell group. The genes and lncRNAs with a difference in the dropout rate of greater than 50% between the two groups of cells were defined as differentially dropout genes (DDGs) and differentially dropout lncRNAs (DDLRs).

Differential expression analysis. We calculated the fold changes in the gene/lncRNA expression values between the two groups of cells and selected genes and lncRNAs with a fold change of greater than two (|logFC|> 1, *p* value < 0.05). Then, we defined these genes and lncRNAs as differentially expressed genes (DEGs) and differentially expressed lncRNAs (DELRs).

Herein, we refer to DDGs and DEGs as differential genes, to DDLRs and DELRs as differential lncRNAs, and to the collective set of differential genes and differential lncRNAs as differential molecules.

Then, we calculated the Spearman correlation coefficient (SCC) between each pair of differential molecules and selected strong correlations with |SCC|> 0.4 and *p* value < 0.05. The differential molecules with strong correlations constituted the differential co-expression network. The absolute values of the Spearman correlation coefficients were used as the edge weights.

### Identification of cell types and candidate cell markers

We used the Markov clustering algorithm (MCL) [34] to cluster the differential co-expression network and identify molecule modules, which we call differential modules herein. MCL is a graph-based, rapidly scalable unsupervised clustering algorithm, and it simulates a random flow to discover the communities in the network. Then, we calculated the average expression value of every differential module as a new feature of the cells. Herein, we call this value the differential module feature of cells.

According to the differential module features, we calculated the Manhattan distance between cell pairs and normalized it by the z-score. Then, we applied DBSCAN to cluster the cells, and each cluster was considered a cell type.

For each cell type, we calculated the fold change in the expression level of each gene and lncRNA between cells in that cell type and the remaining cells and selected genes and lncRNAs with a significant difference of at least a fourfold change (|logFC|> 2, *p* value < 0.05) as the candidate cell marker genes and candidate cell marker lncRNAs for that cell type. Herein, the candidate cell marker genes and lncRNAs are collectively referred to as candidate cell markers for a cell type.

## Results

### Feature selection

**Fig. 2:**
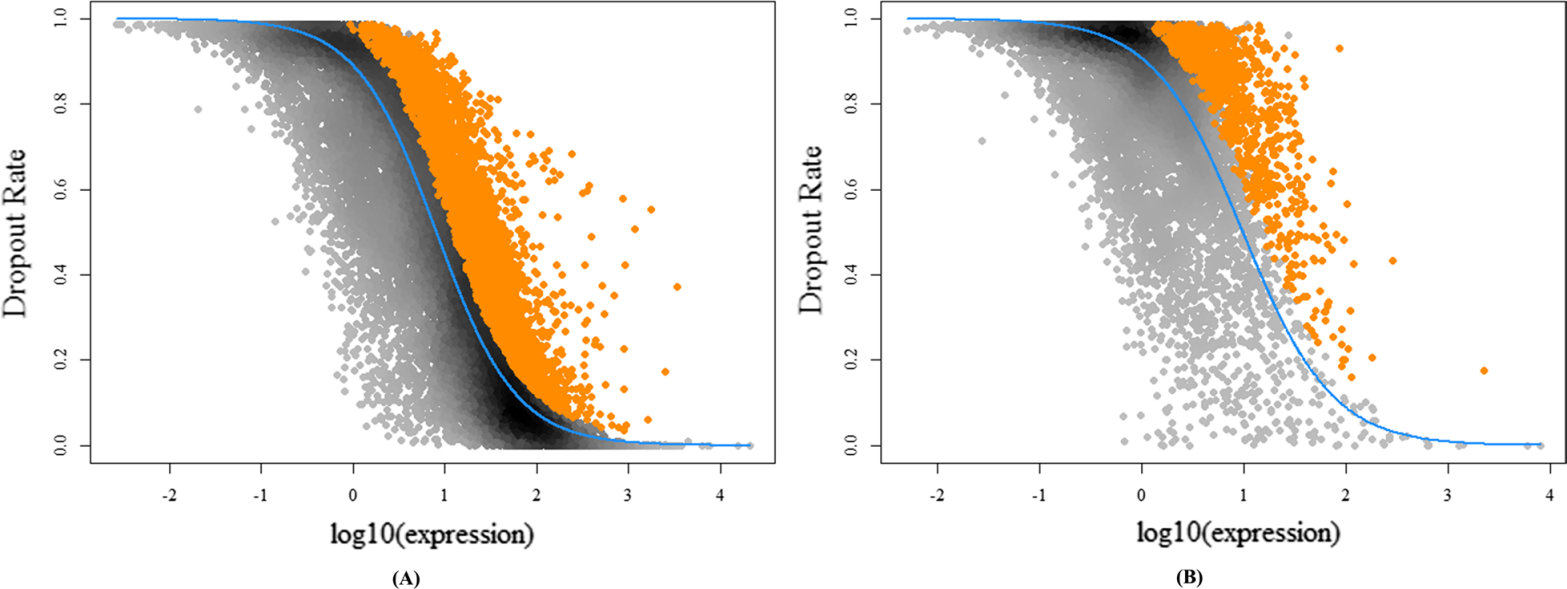
Results of feature selection using M3Drop. The solid blue lines indicate the Michaelis–Menten curves fit to all genes or lncRNAs, and the orange dots indicate the significant genes or lncRNAs with an FDR of < 0.01 in the hypothesis test. **A** Results of feature selection for genes. **B** Results of feature selection for lncRNAs

The results of data preprocessing are shown in Fig. 2(A) and (B). Finally, 3,454 genes and 966 lncRNAs were selected for further analysis. We also performed log transformation on the expression data with a base of 2 and an offset of 1.

### Identification of dropout clusters and differential molecules

The dropout distances between every pair of cells were calculated according to the dropout feature matrix, and the cells were analyzed with DBSCAN [32]. Four cell dropout clusters that individually contained 27, 8, 51 and 45 cells. The result of the dropout clusters was a transition to get the final cell types.

Furthermore, differential dropout analysis and differential expression analysis were performed on different dropout clusters, and the number of differential molecules identified is shown in Table 1. Since some DEGS, DDGs, DELRs and DDLRs may belong to multiple clusters, *Sum* represents the total number of four clusters’ molecules. More differential expressed genes than differential expressed lncRNAs were identified, and the differences in the expression levels were generally greater than the differences in the dropout rates. Differential analysis of all dropout clusters identified 1950 differential expressed genes and 209 differential expressed lncRNAs. The number 1950 means the total number of DEGs and DELRs after deduplication. Similarly, the number 209 means the total number of DDGs and DDLRs after deduplication.

**Table 1:** Number of differential molecules in the dropout clusters, including the number of differential expressed genes/lncRNAs in each dropout cluster and the number of differential molecules in all dropout clusters

Dropout cluster		1	2	3	4	Sum
Differential expressed genes	DEGs	805	6	1185	1067	1940
	DDGs	17	46	80	143	263
Differential expressed lncRNAs	DELRs	5	2	119	146	207
	DDLRs	0	4	7	7	16

### Analysis of the differential co-expression network

For the 2159 differential expressed molecules (1950 differential expressed genes and 209 expressed differential lncRNAs) obtained from the differential analysis, the Spearman correlation coefficient between each pair of differential molecules was calculated with cut-off criteria of |SCC|> 0.4 and *p* value < 0.05. We obtained 48,940 strong correlations among differential expressed molecules, specifically, 17,908 positive correlations and 31,032 negative correlations. The resulting differential co-expression network was an undirected and weighted network consisting of 892 nodes and 48,940 edges.

The differential co-expression network was a scale-free network with a power law node degree distribution. The protein-coding gene HLA-DRA was a hub node in the network, with a degree of 484. GeneCards [35] shows that *HLA-DRA* is a protein-coding gene whose main function is to bind to peptides produced by antigens in the endocytosis of antigen-presenting cells (APCs) and display them on the cell surface for recognition by CD4+ T cells.

The edge weights in the differential co-expression network were calculated as the absolute values of the Spearman correlation coefficients. Among the connected nodes, the protein-coding gene *PHACTR1* had the strongest correlation with the lncRNA *AL008729.2*, with the Spearman correlation coefficient of 0.96.

### Identification of differential modules and cell types

**Fig. 3:**
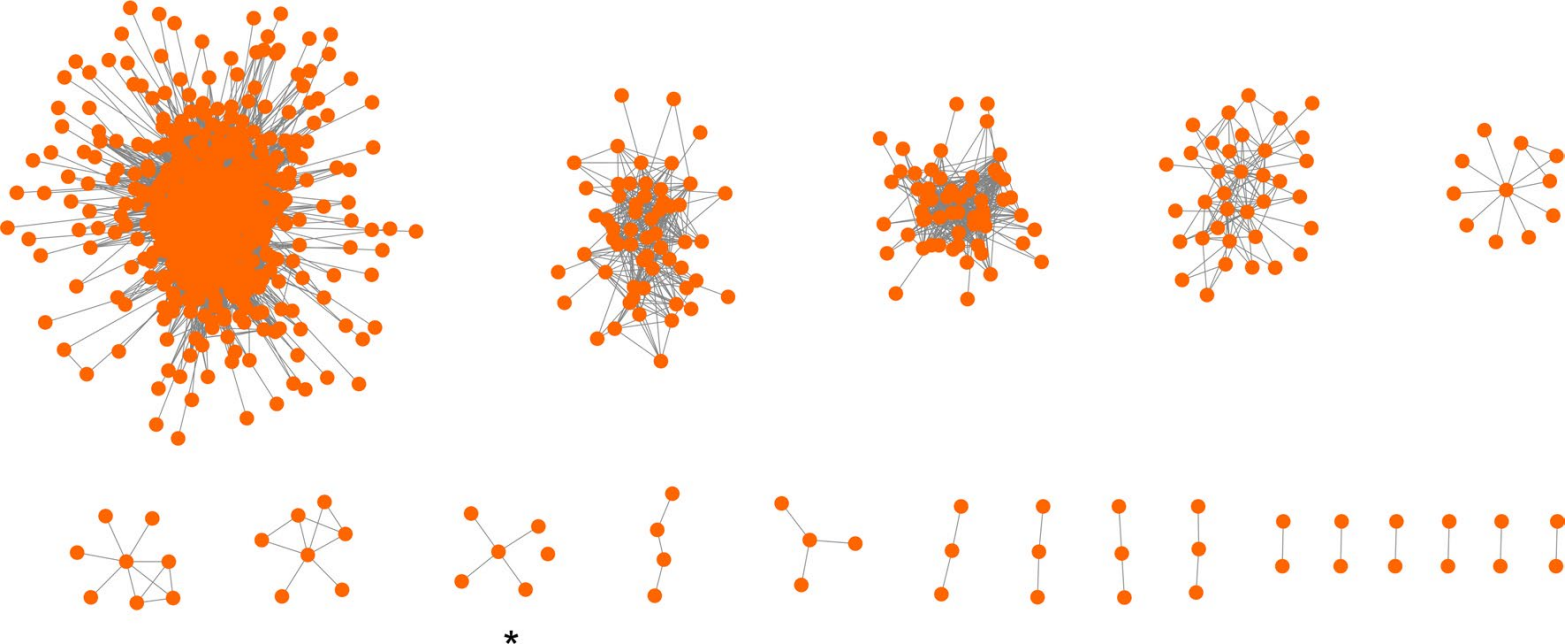
Structure of subnetworks of differential modules. Each connected network is a differential module, except for the module marked by *, which includes a separate node

The differential molecules in the differential co-expression network were further divided by MCL with the inflation parameter set at 2.5. Twenty differential modules were identified. For each identified differential module, we extracted the sub-network from the differential co-expression network (Fig. 3).

Then, we calculated the differential module features of cells and used DBSCAN to identify cell types. A total of eight cell types were identified and are denoted by the letters A–H. The number of cells of each cell type is shown in Table 2. Cell type B contained significantly more cells than the other seven cell types, each of which contained a relatively small number of cells. In addition, 100 cells did not belong to any cell type; we then combined all of these cells into a distinct cell type named cell type 0.

**Table 2:** Number of cells in each cell type

Cell type	A	B	C	D	E	F	G	H
Number of cells	10	121	8	7	11	22	22	6

### Analysis of cell similarity in cell types

**Fig. 4:**
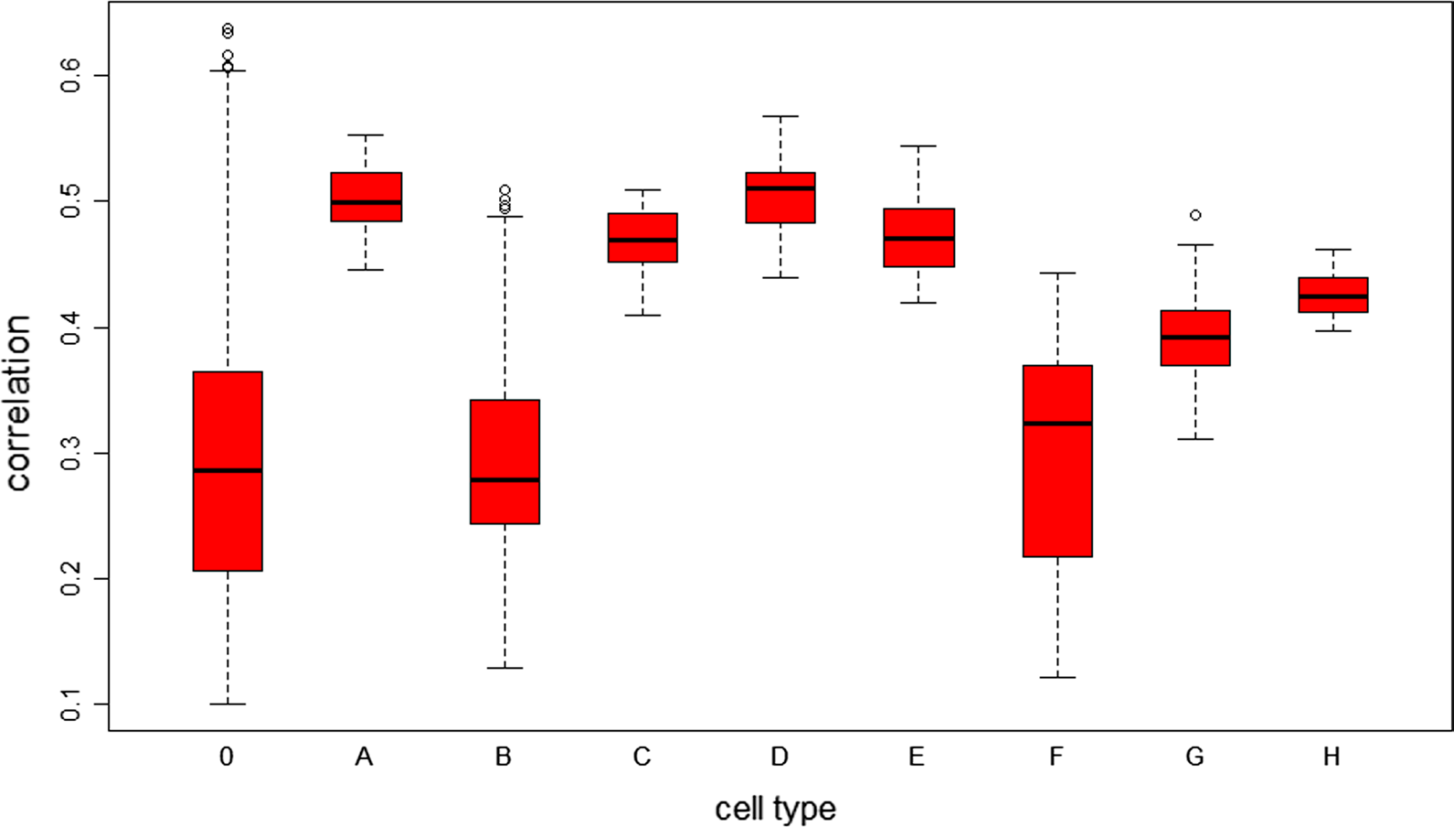
Similarity of molecular expression patterns in each cell type. The Spearman correlation coefficients for the gene and lncRNA expression levels in all cells in each cell type were calculated, and all correlation coefficients were significant

The Spearman correlation coefficient between each pair of cells in the same cell type was calculated according to the expression values of genes and lncRNAs to indicate the similarity of the expression patterns between the two cells. The boxplot of the similarity between cells in each cell type is shown in Fig. 4.

These results showed that the expression patterns of any two cells in the same cell type were positively correlated with significant *p* values. Figure 4 indicates that the average similarity of cells in all cell types except cell type B was significantly higher than that in cell type 0. In particular, in cell types A, C, D, and E, the lowest correlation coefficient between two cells was greater than 0.4, and the correlation coefficients between all cells in cell types G and H were greater than 0.3. In addition, in cell type 0, the correlation coefficients between cells had a large span and a low average value, consistent with the experimental results indicating that the cells did not belong to the same cell type.

### Differential analysis between cell types

Furthermore, the R package Limma [36] was used to calculate the fold changes in expression levels, and the significantly differential genes and lncRNAs with at least a fourfold change in expression were selected as candidate cell markers. The number of candidate cell markers obtained are shown in Table 3. More than 200 candidate cell markers were identified for cell types A, E, G, and H, indicating significant differences between cells in these cell types and other cell types.

**Table 3:** Number of candidate cell markers for each cell type, including candidate cell marker genes and candidate cell marker lncRNAs

Cell type	0	A	B	C	D	E	F	G	H
Number of candidate cell marker genes	0	213	79	20	96	207	92	216	224
Number of candidate cell marker lncRNAs	0	23	5	7	15	37	0	6	38
Number of candidate cell markers	0	236	84	27	111	244	92	222	262

We analyzed regulation directions of the candidate cell markers for each cell type. In cell types A, C, and D, all candidate cell markers were upregulated (logFC > 0). In cell type E, 3 candidate cell marker genes were downregulated (logFC < 0), and the remaining candidate cell markers were upregulated. In cell type F, only one candidate cell marker gene was upregulated, and the rest were downregulated. Candidate cell markers in other cell types were upregulated and downregulated in different patterns.

In addition, in cell type 0, no significant similarity was observed among the expression patterns of the cells, and no candidate cell markers were found, which verified the reliability of the results for the eight identified cell types.

### Prediction of candidate cell markers in melanoma

**Fig. 5:**
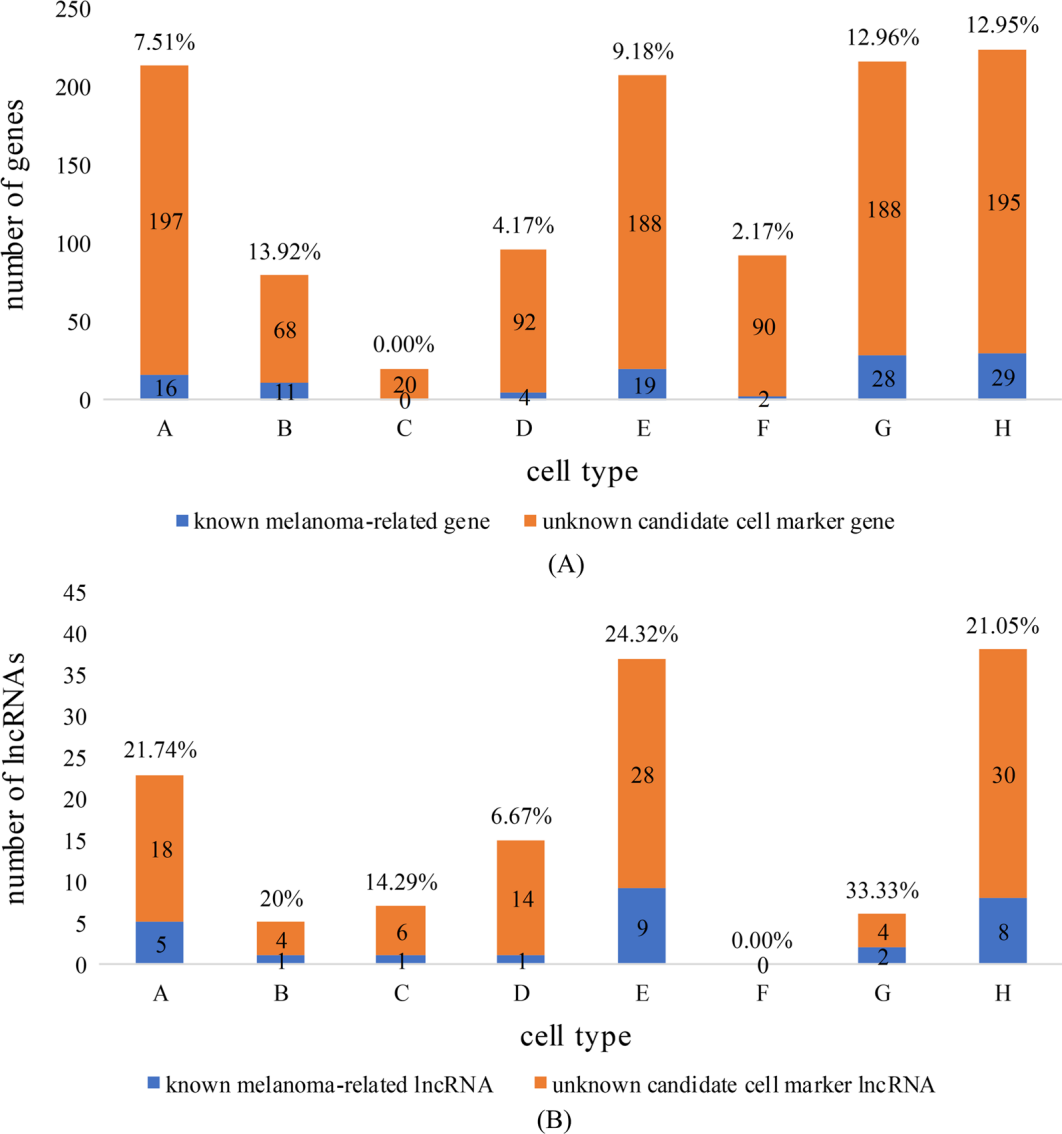
Relationships between candidate cell markers for each cell type and known melanoma-related biomolecules. **A** Relationships between candidate cell marker genes and known melanoma-related genes, including the numbers and percentages of known melanoma-related genes. **B** Relationships between candidate cell marker lncRNAs and known melanoma-related lncRNAs, including the numbers and percentages of known melanoma-related lncRNAs

To verify the relationships between the cell types and melanoma, we collated 580 known melanoma-related genes and 2719 known melanoma-related lncRNAs from multiple databases and published research results and compared them with candidate cell markers in each cell type (see the Materials and Methods for details). The results are shown in Fig. 5.

Figure 5(A) indicates that except for cell type C, which had only 20 candidate cell marker genes, all cell types had known melanoma-related genes among the candidate cell marker genes. The candidate cell markers for cell types H and G included 29 and 28 known melanoma-related genes, respectively. In addition, in cell types B, G, and H, known melanoma-related genes accounted for more than 10% of all candidate cell marker genes.

Figure 5(B) indicates that 9, 8, and 5 candidate cell marker lncRNAs in cell types E, H, and A, respectively, were known melanoma-related lncRNAs and that known melanoma-related lncRNAs accounted for the largest percentage of candidate cell marker lncRNAs in cell type G (33.33%).

The above analysis of the correlations between eight cell types and melanoma indicated that candidate cell markers in cell types A, B, E, G, and H, especially cell types G and H, were strongly related to melanoma.

### Analysis of known melanoma cell markers

Four known melanoma cell marker genes, *MITF*, *MLANA*, *PMEL* and *TYR*, were obtained from CellMarker [29]. Then, we investigated whether these four known melanoma cell markers appeared among the candidate cell markers for each cell type.

All four known melanoma cell markers were candidate cell marker genes for cell type H, and *MLANA*, *PMEL*, and *TYR* were candidate cell marker genes for cell type G. In addition, the protein-coding gene *PMEL* was a candidate cell marker gene for five cell types (all cell types except C, D, and F). *PMEL* plays a central role in the biogenesis of melanosomes and participates in the maturation of melanosomes from stage I to stage II [37, 38]. According to GeneCards, *PMEL* is associated with the incidence of various melanomas, such as skin melanoma, gallbladder melanoma, and melanoma in congenital melanocytic nevus.

*PMEL*, also known as premelanosome protein gene, participates in the maturation process of melanosomes from phase I to phase II, and plays a central role in melanogenesis [37, 38]. *MITF* plays a role in multiple activity levels that determine the fate of melanoma cells. Melanoma cells that highly express *MITF* can differentiate or proliferate. Stem cell-like or invasive potential can cause low *MITF* activity. And long-term *MITF* inhibition will drive cell senescence [39]. *MITF* up- or down-regulation modulates *MLANA* expression in parallel directions at both mRNA and protein levels. As a target gene for melanocyte restriction, *MLANA* may provide an opportunity to study whether their melanocyte restriction expression is produced by the unique activity of the *MITF* melanocyte isotype, or whether other transcription factors may contribute (together with *MITF*) give melanocyte-specific expression [40]. *TYR*, *TYRP1* and downstream enzymes metabolize tyrosine to melanin [41].

These results showed that the candidate cell markers for cell types G and H were closely related to the known melanoma cell markers and that other unknown candidate cell markers in these two cell types could be potential driver biomolecules for melanoma.

### Enrichment analysis of key cell types

The previous analysis of the eight cell types indicated that cell types G and H were highly correlated with melanoma and that the cells belonging to these cell types had similar expression patterns and were significantly different from the cells not belonging to these cell types. We defined these two cell types as the key cell types associated with melanoma and further used the R package clusterProfiler [42] to perform Gene Ontology (GO) functional and Kyoto Encyclopedia of Genes and Genomes (KEGG) pathway enrichment analyses of their candidate cell marker genes to explore the pathogenesis of melanoma.

We performed GO functional enrichment analysis, including analysis of biological process (BP), molecular function (MF) and cellular component (CC) terms, and focused on GO terms with an adjusted p-value (p.adjust) of < 0.01. We obtained 113 and 95 GO terms related to cell types G and H, respectively; the top three GO terms in the BP, MF and CC aspects are shown in Tables 4 and 5. Comparison of the results revealed that cell type G was related to the mitotic process of cells, while cell type H was more strongly related to activities of the major histocompatibility complex (MHC).

**Table 4:** Gene Ontology (GO) functional enrichment analysis of candidate cell marker genes for cell type G. The top 3 GO terms in the biological process (BP), molecular function (MF) and cellular component (CC) aspects are listed (p.adjust < 0.01)

Aspect	GO ID	Descriptions	p.adjust	Count
	GO:0140014	Mitotic nuclear division	7.11E−06	17
BP	GO:0048285	Organelle fission	2.83E−05	20
	GO:0000280	Nuclear division	2.83E−05	19
	GO:0023026	MHC class II protein complex binding	0.001115	4
MF	GO:0023023	MHC protein complex binding	0.005092	4
	GO:0050786	RAGE receptor binding	0.006432	3
	GO:0042613	MHC class II protein complex	1.47E−05	6
CC	GO:0000793	Condensed chromosome	2.83E−05	14
	GO:0098687	Chromosomal region	3.37E−05	17

**Table 5:** Gene Ontology (GO) functional enrichment analysis of candidate cell marker genes for cell type H. The top 3 GO terms in the biological process (BP), molecular function (MF) and cellular component (CC) aspects are listed (p.adjust < 0.01)

Aspect	GO ID	Descriptions	p.adjust	Count
	GO:0140014	Mitotic nuclear division	7.59E−06	17
BP	GO:0019886	Antigen processing and presentation of exogenous peptide antigen via MHC class II	7.83E−06	11
	GO:0002495	Antigen processing and presentation of peptide antigen via MHC class II	8.00E−06	11
	GO:0023026	MHC class II protein complex binding	0.000107	5
MF	GO:0023023	MHC protein complex binding	0.000724	5
	GO:0042605	Peptide antigen binding	0.001458	5
	GO:0042613	MHC class II protein complex	6.05E−07	7
CC	GO:0042611	MHC protein complex	7.81E−06	7
	GO:0030669	Clathrin-coated endocytic vesicle membrane	1.06E−05	8

In addition, some enriched GO terms in the two key cell types were associated with melanin and melanosomes (p.adjust < 0.05), as shown in Table 6, supporting the reliability of the identification of the two key cell types.

**Table 6:** The GO terms related to melanin and melanosomes in the GO enrichment (p.adjust < 0.05). "–" means that this term did not appear in the enrichment results for this cell type

Aspects	GO ID	Descriptions	p.adjust in cell type G	p.adjust in cell type H
BP	GO:0042438	Melanin biosynthetic process	2.83E−05	0.000325
BP	GO:0006582	Melanin metabolic process	2.83E−05	0.000404
CC	GO:0033162	Melanosome membrane	4.27E−05	0.001092
CC	GO:0042470	Melanosome	0.000143	0.001458
BP	GO:0030318	Melanocyte differentiation	0.005577	0.049285
BP	GO:0032402	Melanosome transport	0.03374	–
BP	GO:0032401	Establishment of melanosome localization	0.03712	–
BP	GO:0032400	Melanosome localization	0.044045	–
BP	GO:0032438	Melanosome organization	0.044045	0.047491

**Fig. 6:**
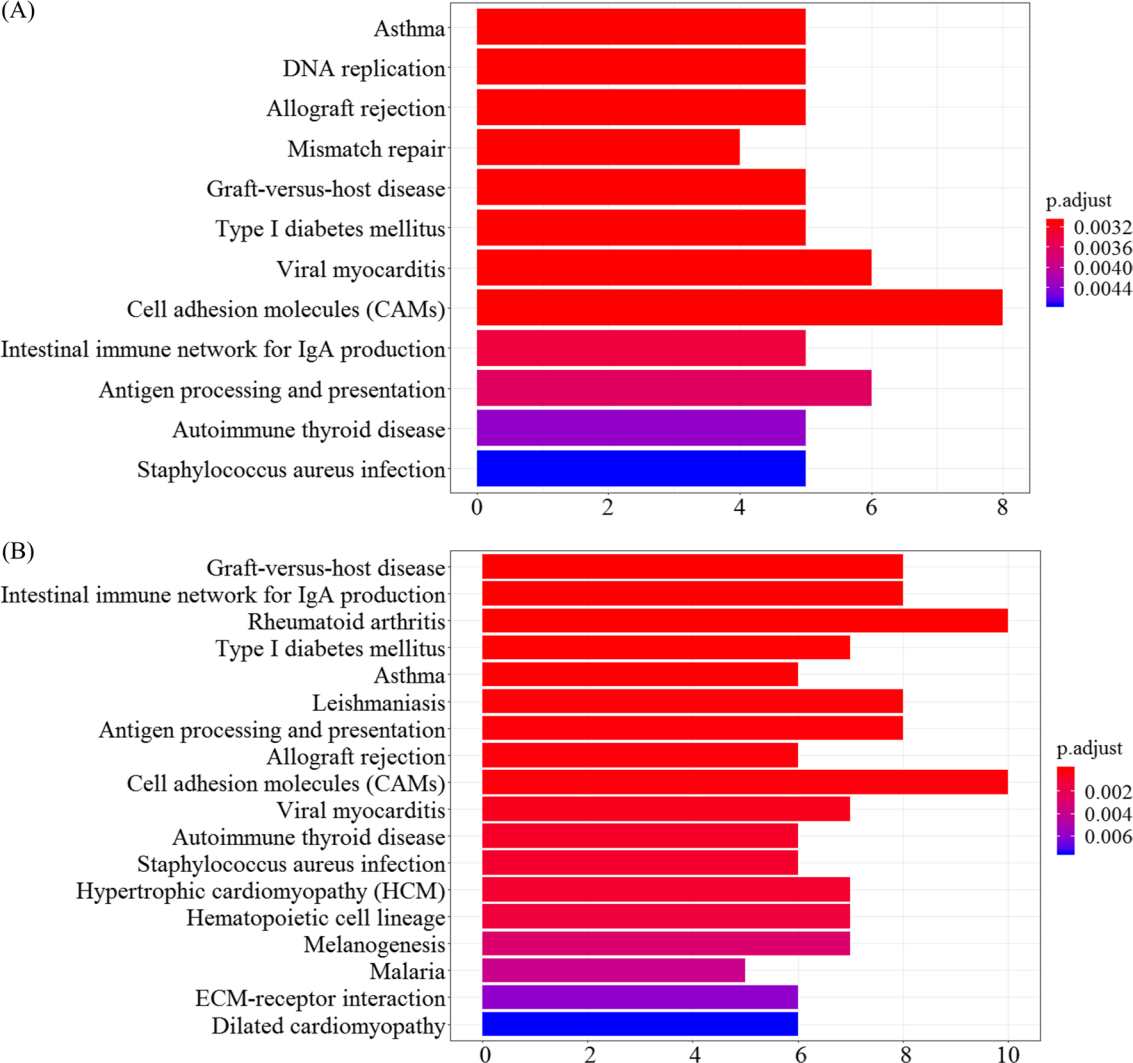
KEGG pathway enrichment analysis of candidate cell marker genes for the two key cell types (p.adjust < 0.01). **A** Cell type G. **B** Cell type H

KEGG pathway enrichment analysis of candidate cell marker genes in the two cell types are shown in Fig. 6(A) and (B). A total of 12 and 18 significantly enriched pathways (p.adjust < 0.01) were related to cell types G and H, respectively.

The two cell types shared 10 enrichment pathways, many of which were related to immune response processes, such as antigen processing and presentation, allograft rejection, and autoimmune thyroid disease. Cell type G was related to DNA replication and mismatch repair pathways. Cell type H was related to melanogenesis and ten diseases, namely, graft-versus-host disease, rheumatoid arthritis, type I diabetes mellitus, asthma, leishmaniosis, viral myocarditis, autoimmune thyroid disease, hypertrophic cardiomyopathy, malaria, and dilated cardiomyopathy.

The functional and pathway enrichment analysis indicated that the two key cell types were strongly related to the biological activities of melanin and melanosomes. In addition, candidate cell marker genes for cell type G were significantly enriched in mitosis-related biological activities, and cell type H was associated with the occurrence of ten diseases.

## Discussion

scRNA-seq technology allows researchers to study biomolecules at the single-cell level so that the molecular mechanisms of some complex diseases, such as cancer, can be studied and analyzed at a single-cell resolution.

In this paper, considering the characteristics of the scRNA-seq data, we proposed a framework for cell type identification and applied it to a single-cell melanoma dataset. Two key cell types related to melanoma were identified, and the molecular mechanisms of melanoma were analyzed at the single-cell level.

First, making full use of the dropout information in the gene expression data, we identified four different dropout clusters and found that the expression levels of the protein-coding gene REXO2 differed significantly among the four dropout clusters.

Then, MCL was performed on the differential co-expression network, and 20 differential modules were identified. Then, eight cell types were identified by using the differential modules as new cell features. Our analysis identified strong correlations among cells in each cell type, with similar expression patterns, and revealed significant differences among cells of different cell types. In addition, we found that each cell type showed a different extent of association with melanoma.

Finally, we defined cell types G and H as the key cell types associated with melanoma and found that both of these key cell types were related to melanosomes and melanin and were highly correlated with the biological activities of MHC molecules. In addition, cell type G was related to cell mitosis, and cell type H was related to ten diseases.

In summary, by identifying cell types of melanoma cancer cells and further analyzing all cell types, we distinguished two key cell types that are highly related to melanoma, providing a key insight for the future direction of melanoma research. In addition, candidate cell markers for the two key cell types can be focused on as potential therapeutic targets for melanoma. Furthermore, the computational framework proposed in this paper is not limited to melanoma and can be extended to the pathological study of other cancers or complex diseases.

## Conclusion

We proposed a computational framework for cell type identification that fully considers cell dropout characteristics. This method was applied to single-cell RNA-seq data of melanoma, and eight cell types were identified. Enrichment analysis of the candidate cell marker genes for the two key cell types showed that both key cell types were closely related to the physiological activities of the MHC. Through identification and analysis of key melanoma-related cell types, we explored the molecular mechanism of melanoma, providing insight into melanoma research. Moreover, the candidate cell markers for the two key cell types are potential therapeutic targets for melanoma.

## Data Availability

The data underlying this article are available in CancerSEA at http://biocc.hrbmu.edu.cn/CancerSEA/goDownload, and can be accessed with EXP0072.

## Abbreviations

RNA-seq: RNA sequencing
DEGs: Differentially expressed genes
scRNA-seq: Single-cell RNA sequencing
CIDR: Clustering through imputation and dimensionality reduction
MAGIC: Markov affinity-based graph imputation of cells
DBSCAN: Density-based spatial application of applications with noise
DDGs: Differentially dropout genes
DDLRs: Differentially dropout lncRNAs
DELRs: Differentially expressed lncRNAs
SCC: Spearman correlation coefficient
MCL: Markov clustering algorithm
GO: Gene ontology
KEGG: Kyoto encyclopedia of genes and genomes
BP: Biological process
MF: Molecular function
CC: Cellular component
MHC: Major histocompatibility complex
